# Integrated fractal clustering and inversion of induced polarization data for concealed gold exploration in Kabudan area NE Iran

**DOI:** 10.1038/s41598-026-38850-8

**Published:** 2026-02-12

**Authors:** Seyed Mohammad Sadatian Jouybari, Ahmad Afshar, Hamidreza Ramazi, Shahriyar Asadi, Morteza Hasiri

**Affiliations:** 1 Faculty of Mining Engineering, Amirkabir University of Technology, Tehran, Iran; 2 Kharazmi University, Tehran, Iran

**Keywords:** Fractal, Chargeability, Electrical resistivity, Clustering, Gold mineralization, Kabudan, Iran, Geophysics, Economic geology, Mineralogy

## Abstract

The identification and delineation of concealed mineralized zones in settings with weak or overlapping anomalies remains a critical challenge. Conventional geophysical methods provide limited resolution and reliability in such conditions. To overcome this limitation, this study introduces a systematic framework that integrates fractal clustering and geophysical inversion to enhance the accuracy of mineral exploration. Induced polarization (IP) chargeability data, acquired using a rectangular array at the Kabudan gold prospect in northeastern Iran—an area characterized by the Lack of outcrops and surface indications of mineralization—were analyzed using four well-established fractal models: Concentration–Area (C–A), Concentration–Perimeter (C–P), Concentration–Number (C–N), and Number–Size (N–S). To quantitatively evaluate the performance of each model, four statistical validation indices were employed: Silhouette, Davies–Bouldin, Calinski–Harabasz, and cluster stability. Among these models, The C–P fractal model exhibited the highest clustering quality, with the highest Silhouette index (closest to 1 among the models), the lowest Davies–Bouldin index, the highest Calinski–Harabasz index, and the lowest Silhouette index standard deviation (highest cluster stability). To verify the subsurface continuity of the identified anomalies, Four geoelectrical profiles were acquired over the anomalous zones, and two-dimensional (2D) inversion of the induced polarization (IP) and resistivity data was performed. The data were subsequently modeled, and the corresponding cross-sections were generated to illustrate the subsurface variations. The inverted sections revealed coherent chargeable structures that closely corresponded to the clusters derived from the fractal models. The results were further assessed and validated using borehole data, where the correspondence between a high-grade gold-bearing sulfide zone and the anomalies delineated in the profiles confirmed the reliability and accuracy of the interpretations. Overall, the proposed integration of fractal clustering, geophysical inversion, and statistical validation not only enhances the interpretability of subsurface data under complex geological conditions but also provides a scalable and transferable framework for next-generation mineral exploration.

## Introduction

 The primary objective of mineral exploration is to discover new mineral-rich zones within targeted regions^1^. Geophysical methods play a crucial role in investigating subsurface conditions for identifying concealed mineral deposits. As non-destructive approaches, they are widely applied in various exploratory, engineering, and environmental studies, contributing significantly to the optimization of both cost and time^2,3^. The Induced Polarization (IP) and Electrical Resistivity (ER) methods are among the fundamental tools in the exploration of metallic ore deposits, particularly sulfide mineralizations^4^. The combination of these methods with advanced inversion and data classification techniques enables the delineation of mineralized zones, even in areas with thick overburden or complex geology^5,6^. In mineral exploration, the main objective of geophysical surveys is to obtain a better understanding of the geological framework of the study area, delineate anomalies, and map subsurface geological structures^7^. Multi‑modal geophysical surveys have proven effective in enhancing subsurface characterization and reducing interpretation uncertainties^8,9^.

Fractal geometry, first introduced by Mandelbrot in the 1980s, has become a powerful framework for understanding and modeling the inherent complexity of natural systems It has been extensively applied across the geosciences, not only for analyzing intricate geological structures but also in diverse domains such as engineering, economic geology, geophysical analysis, and geochemical anomaly separation to identify zones of mineral concentration^10,11^.

Fractal geometry defines non-integer dimensions—known as fractal dimensions—which are widely used for quantifying the complexity of patterns and distributions in geoscience data^12^. These methods offer several significant advantages, the most important of which is their ability to delineate and characterize the geometric structure of geological zones based on the spatial distribution of relevant parameters^13–15^.

Traditional statistical approaches primarily distinguish between anomalous and background classes but fail to account for the spatial distribution and autocorrelation structure of the data. In contrast, geostatistical interpolation models can analyze spatial structures by incorporating spatial dependencies when interpolating and identifying trends. However, the major limitation of these interpolation methods is their tendency to oversmooth or flatten anomalies, which may obscure subtle features or cause weak anomaly zones to merge with areas of high background values. This critical issue can lead to the omission of important exploration targets—an increasingly common challenge in mineral prospectivity mapping, particularly under complex geological conditions^16,17^.

Fractal and multifractal approaches, in contrast, account for both the geometric characteristics of anomalies and the spatial distribution of the underlying data. Moreover, they utilize all available measurements without modification, enabling more accurate discrimination between anomaly classes and background signals^18,19^. Accordingly, various fractal analysis methods—such as concentration–area, concentration–perimeter, number–size, and concentration–number—have been developed and increasingly adopted for anomaly separation in both geochemical and geophysical datasets.

Given that the primary objective in geophysical surveys is to distinguish and isolate anomalous zones from background regions, the application of advanced data analysis techniques can play a significant role in enhancing interpretation processes. In this context, fractal methods, owing to their capability to describe complex patterns, can provide a more accurate analysis of geophysical data^20^ Recent studies have demonstrated that fractal-based approaches, especially when integrated with clustering algorithms and statistical validation indices, can significantly improve the detection and classification of geophysical anomalies, facilitating the prioritization of exploration targets^6,21^.

Studies by Ferdows and Ramazi (2015), Akbari et al. (2023), and Nazih et al. (2025), which employed fractal and multifractal approaches in the processing and interpretation of geophysical data, have demonstrated significant success in improving the identification and delineation of subsurface anomalies^1,22,23^.

Despite numerous studies utilizing these methods in geochemical exploration, comprehensive comparative analysis of fractal models in geophysical data remains limited, especially under challenging field conditions. Furthermore, a thorough and systematic evaluation of the performance of widely adopted fractal analysis methods through numerical validation has not yet been reported, highlighting a significant gap in current geophysical research. Therefore, evaluating the discrimination power of different fractal models on real geophysical datasets can provide valuable insights for mineral exploration in complex environments.

 The remainder of this paper is organized as follows: Section "Geological setting" outlines the geological setting of the Kabudan gold prospect within the Taknar structural zone, emphasizing lithological units, alteration types, and their relevance to induced polarization responses. Section "Materials and methods" describes the geophysical surveys and analytical methodology, including the four fractal clustering models—Concentration–Area (C–A), Concentration–Perimeter (C–P), Concentration–Number (C–N), and Number–Size (N–S)—together with the statistical validation metrics used. Section "Results" presents the results of fractal classification, geoelectrical inversion, and borehole validation. In Section "Discussion", the findings are discussed in relation to comparative model performance and mineral exploration implications in complex geological settings. Finally, Section "Conclusion" concludes the study, summarizing the key contributions of the integrated fractal–geophysical workflow and suggesting avenues for future research.

## Geological setting

**Fig. 1:**
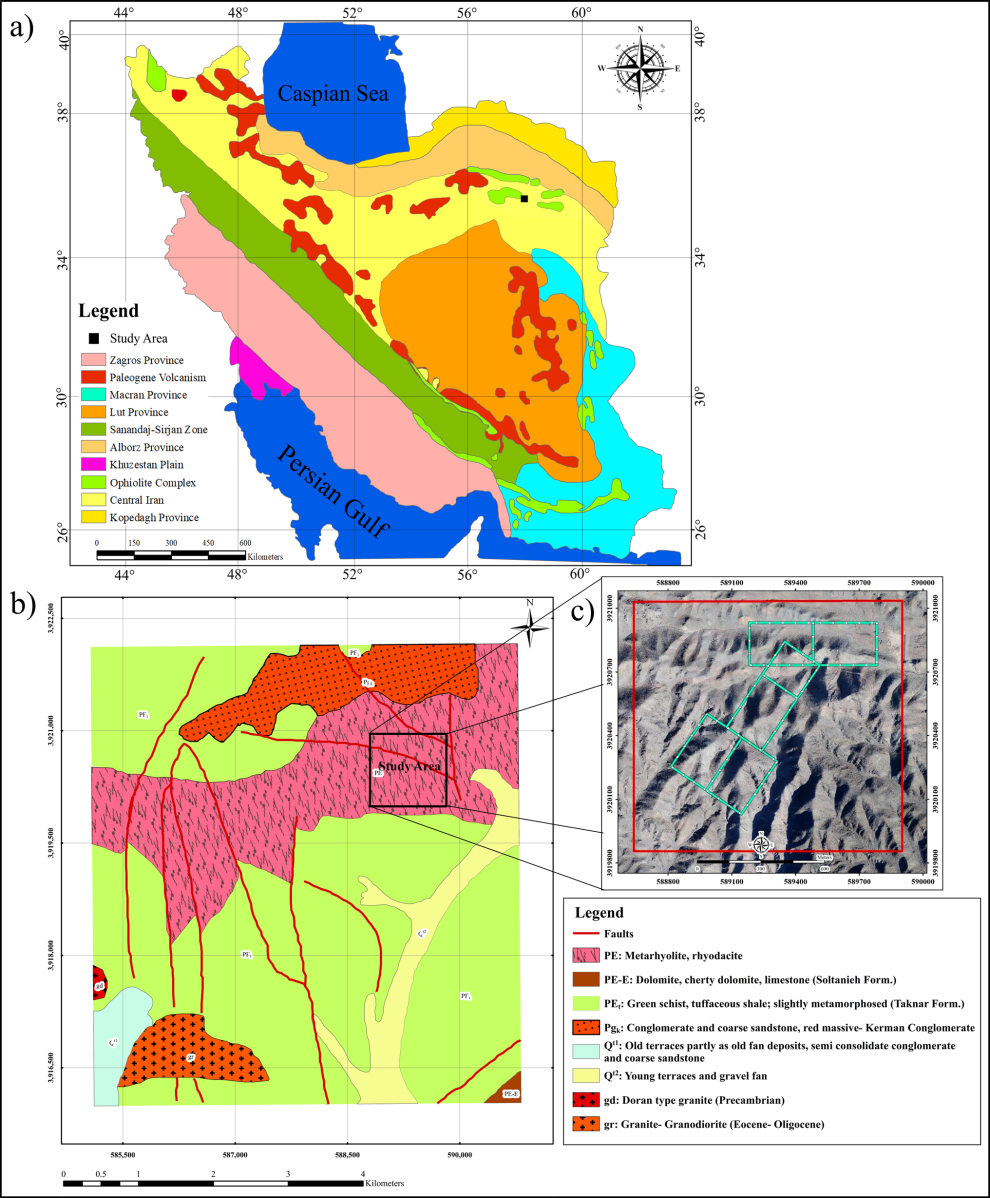
Study area location within the structural zones of Iran (**a**), geological map of the Kabudan area, north of Bardaskan, extracted from the 1:100,000 Bardaskan geological map (**b**), and layout of the rectangular arrays surveyed in the area (**c**). All maps were generated using QGIS version 3.28.3 (https://qgis.org). Geological map data were digitized and interpreted by the authors based on the original source.

The Kabudan mineralization complex is located north of Bardaskan, within the Taknar structural zone in northeastern Iran. Based on the tectonic subdivision of Iran, the Taknar zone forms part of the Central Iranian structural domain (Fig. 1a–c). The maps in Fig. 1 were prepared using QGIS version 3.28.3 (https://qgis.org) from digitized and interpreted data of the 1:100,000 Bardaskan geological map published by the Geological Survey of Iran^24,25^. From a geotectonic perspective, this zone occupies an intermediate position between the Lut Block to the south and the Sabzevar zone to the north, bounded by the Doruneh (Great Kavir) Fault in the south and the Taknar (Rivash) Fault in the north^26,27^.

The Taknar Formation, also known as the Taknar Erosional Window, represents a narrow, uplifted segment of the Precambrian–Paleozoic basement, unconformably overlain by Mesozoic–Cenozoic strata, and is interpreted as a remnant of an infertile Precambrian continental rift. It has undergone low-grade metamorphism corresponding to the green schist facies^28,29^. Lithologically, the formation comprises mica schists, metamorphosed sandstones, and metavolcanic rocks, including meta-rhyolite, meta-dacite, and meta-rhyodacite. The mica schist unit consists of sericite schist, chlorite schist, and chlorite–sericite schist, with protoliths most likely being quartz-rich pelitic sediments and, in some cases, fine-grained sandstones^30^.

Magmatic and tectonic activities during the Precambrian led to the emplacement of a suite of intrusive bodies with compositions ranging from granite–granodiorite to meta diabase and meta gabbro–diorite within the region^28,30^. These rock units represent part of the uplifted Precambrian to Paleozoic basement of Central Iran, which is overlain by Mesozoic–Cenozoic volcanic–sedimentary sequences^31^. The rocks of this zone, which underwent folding, metamorphism, and faulting accompanied by thrusting during the early tectonic stages, have experienced successive episodes of predominantly brittle deformation throughout geological time. The most prominent structural features are right-lateral strike-slip faults trending northeast–southwest^32^.

In general, mineralization in the Taknar Formation occurs along the contact zones between acid metatrophic and metariolitic rocks and evolved green schists. The emplacement of diabasic dykes appears to have played a significant thermal role in the development of siliceous–feldspathic sheets within rhyolitic domes. The mineralized zone is geologically extensive and encompasses a variety of volcanic–sedimentary–stratigraphic bands of the bulk sulfide type^29^.

From a mineral potential perspective, the Taknar zone is economically significant due to the abundance of metallic resources, including gold, copper, lead, zinc, silver, and iron, as well as non-metallic commodities such as feldspar, bauxite, construction materials, and industrial clays^31^. The Bardaskan–Kabudan mineralized district forms an integral part of the Taknar metallogenic province. The geological map of the area is presented in Fig. 1b. Within this area, metallic mineralization, particularly gold, predominantly occurs as epithermal and disseminated types^33^. The principal styles of hydrothermal alteration include chloritization, sericitization, and silicification. Major ore minerals consist of pyrite, chalcopyrite, and native gold, which are primarily concentrated within siliceous veins and sericitically altered zones^34^. Several additional epithermal gold prospects and deposits, including Sebandoon, Bijvard, and Damanghor, have also been identified in the northern part of the Kabudan area^35,36^.

The study area lies immediately north of Kabudan village, approximately 15 km north of Bardaskan, in Razavi Khorasan Province. Two primary hydrothermal alteration types have been identified: silicic alteration and iron oxide alteration. Silicic alteration is characterized by pervasive silicification of host rocks, particularly along fractures and vein margins, affecting both rhyolitic volcanic rocks and sericitic schists. Iron oxide alteration is more widespread, typically associated with fault structures or occurring adjacent to diabase dikes, and is distinguished by abundant hematite and limonite, imparting a distinct red and yellow coloration to the altered rocks.

The lithological units intersected in drill cores include metarhyolite, green schist, metamorphosed sandstone, and gabbro–diorite. Mineralized intervals, exhibiting elevated gold grades and coinciding with high-chargeability zones in geophysical inversion models, predominantly contain pyrite, chalcopyrite, and magnetite. Maximum gold concentrations, recorded within sulfide-rich intervals, reach up to 8 ppm Au, confirming a strong correlation between geophysical anomalies and mineralization zones. Figure 1 illustrates the study area’s location within the structural zones of Iran(a), the geological map of the area(b), and the configuration of the rectangular arrays and geoelectric profiles implemented in the field(c).

## Materials and methods

### Geophysical surveys

**Fig. 2:**
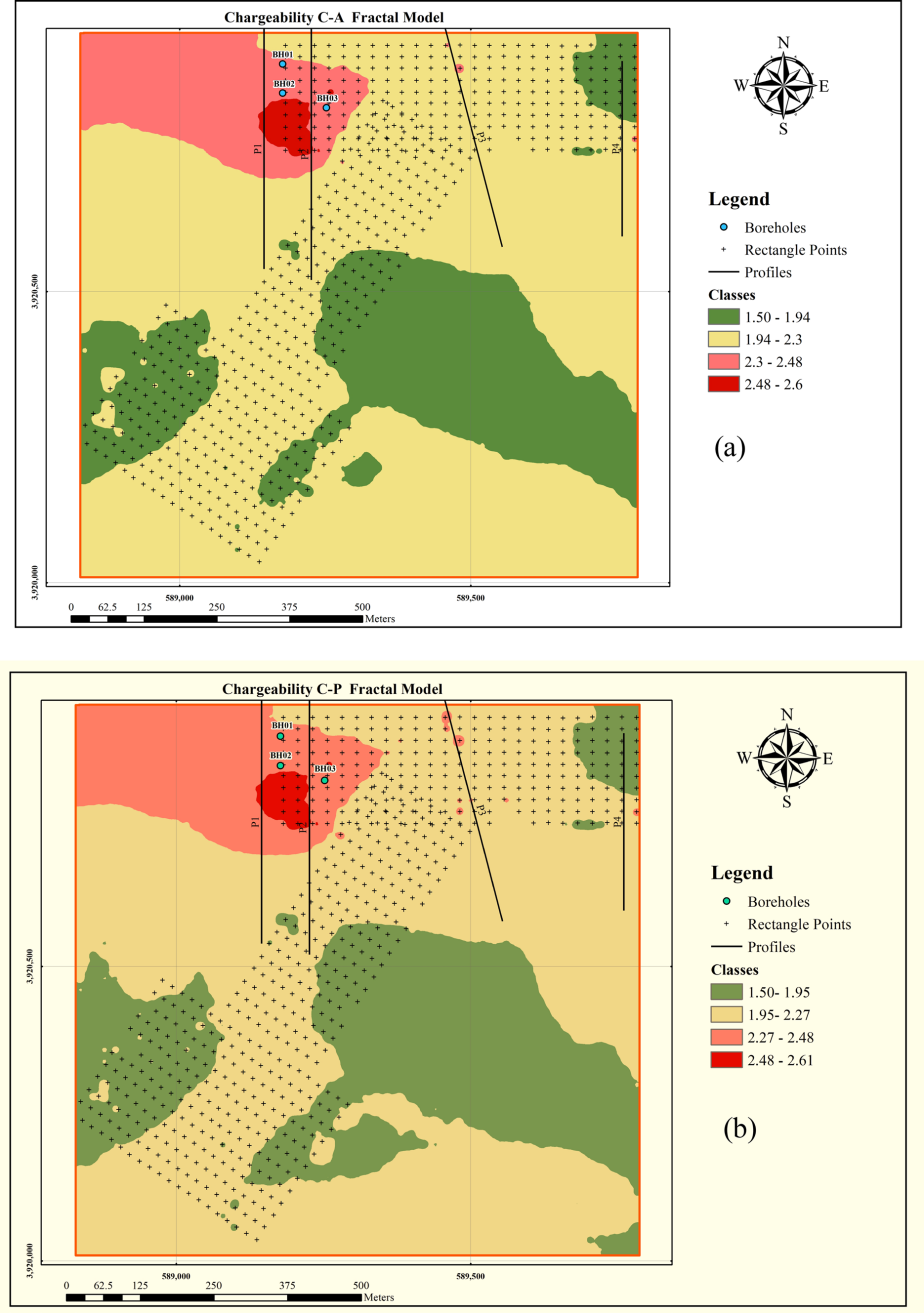

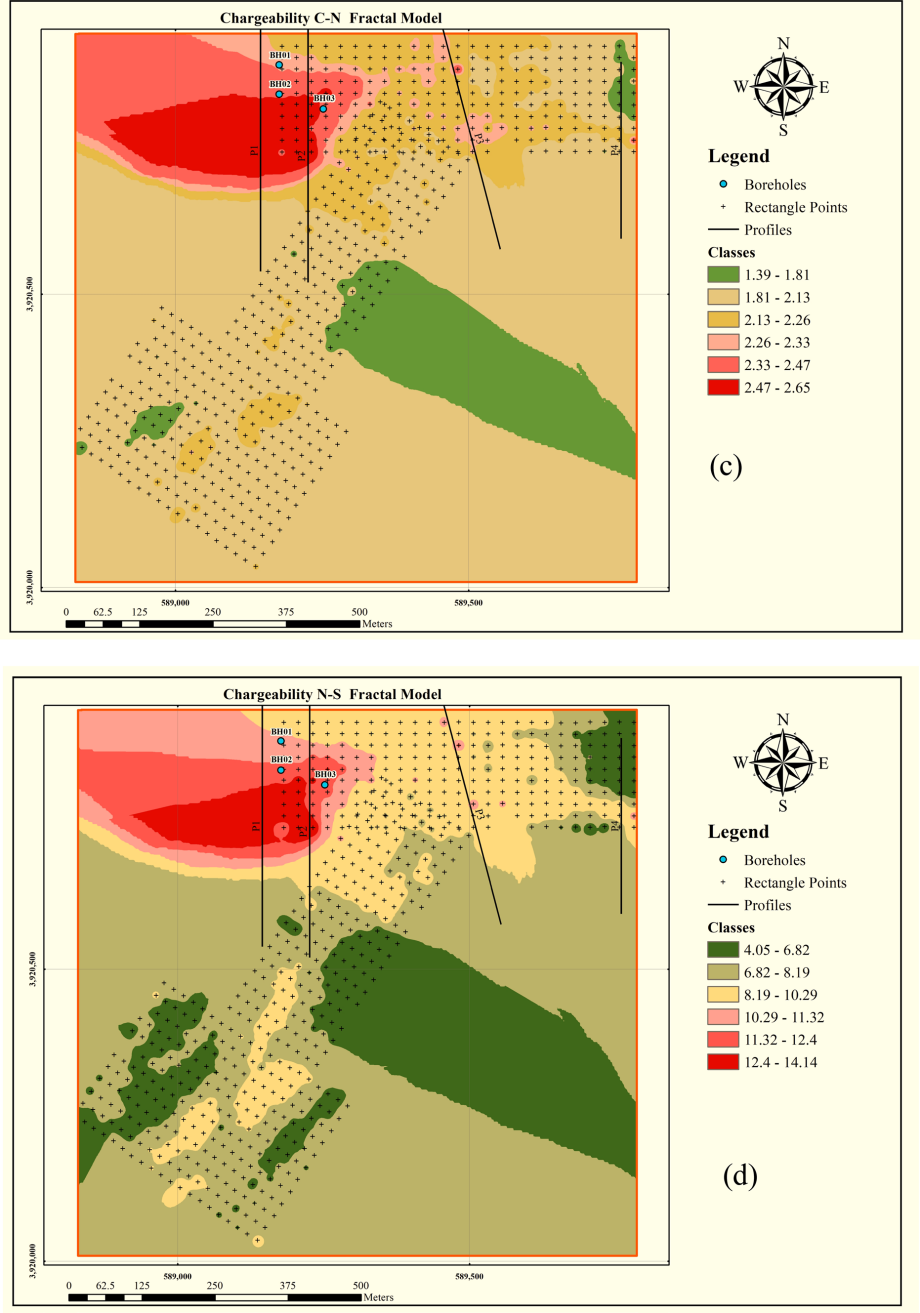
Anomaly maps derived from different fractal models applied to chargeability data: (**a**) Concentration–Area (C–A), (**b**) Concentration–Perimeter (C–P), (**c**) Concentration–Number (C–N), and (**d**) Number–Size (N–S) models. All maps were generated in QGIS version 3.28.3 (https://qgis.org) based on interpreted induced polarization data.

Due to the absence of geological trends and surface mineralization evidence, six rectangular arrays with a current line length of 600 m (AB = 600 m) were acquired to identify the locations of potential anomalies for further investigation. Figure 1c, illustrates the locations of the rectangular arrays acquired within the study area. To classify the data, enhance anomaly discrimination, and delineate the boundaries of chargeability anomalies, the data were clustered using four fractal models: Concentration–Area (C–A), Concentration–Perimeter (C–P), Concentration–Number (C–N), and Number–Size (N–S). To investigate the vertical extent of mineralization and to monitor the depth continuity of the anomalies, four profiles were designed and surveyed for electrical resistivity and induced polarization. The specifications of these profiles are provided in Table 2. The locations of the acquired profiles are presented on the maps in Fig. 2.

**Table 2:** Parameters of the geoelectrical profiles surveyed over chargeability anomalies using the gradient array in the Kabudan exploration area. P1 is the Westernmost and P4 the Easternmost profile.

Profile name	Profile length (m)	Number of points	Array type	Electrode spacing (m)
P1	500	174	Dipole–Dipole	20
P2	500	372	Pole–Dipole	20
P3	500	369	Pole–Dipole	20
P4	300	571	Pole–Dipole	10

### Fractal analysis

Fractals have been widely utilized across a range of scientific fields to facilitate the understanding and analysis of complex processes. Fractal analysis is commonly employed to characterize the spatial and temporal distribution of variables such as element concentrations, with the fractal dimension frequently used to quantitatively describe the degree of complexity and heterogeneity in pattern distributions^37^.

#### Concentration–area model

The concentration–area (C–A) method, originally introduced by Cheng et al. (1994), has not yet gained widespread adoption in the processing of geophysical datasets, but has proven successful in this context^22,38,39^. The fundamental principle of this method is based on the area occupied by each specific value of a concentration parameter within the study area; as the concentration parameter increases, the corresponding area decreases^6^. The general form of the relationship is expressed as:

1 $$A\left( {\rho \le v} \right)\infty \rho ^{{ - a1}} ;A\left( {\rho \ge v} \right)\infty \rho ^{{ - a2}}$$

where A(ρ) represents the area occupied by concentration values exceeding the contour (threshold) ρ, v denotes the threshold value, and − a₁ and − a₂ are the fractal dimensions. Cheng et al. (1994) outlined two principal strategies for calculating A(ρ): (1) the area enclosed by a specific contour line at value ρ on a geochemical map, where primary data are interpolated using a weighted moving average; and (2) the box-counting method, where a grid is superimposed on the study area and A(ρ) is determined by multiplying the number of cells with values greater than ρ by the cell area. For concentrations above a given threshold, the area–concentration relationship typically follows a power-law distribution^40^. Breakpoints observed between linear segments on the log–log plot of A(ρ) versus ρ, along with their corresponding ρ values, can be used as thresholds to distinguish different geochemical populations within the data^39^.

#### Concentration–perimeter model

The concentration–perimeter (C–P) model is conceptually very similar to the C–A fractal method, with its general form given by:

2 $$P\left( {\rho \le v} \right)\infty \rho ^{{ - a1}} ;P\left( {\rho \ge v} \right)\infty \rho ^{{ - a2}}$$

where P(ρ) denotes the perimeter enclosing concentrations greater than the contour value ρ, v is the threshold value, and − a₁ and − a₂ are the corresponding fractal dimensions. In this approach, P(ρ) is defined as the perimeter of the region enclosed by the contour value ρ on an interpolated geochemical map produced using a weighted moving average method^12^.

#### Concentration–number model

Based on the number–size fractal approach, the concentration–number (C–N) fractal model was proposed by Hassanpour and Afzal (2013) for the analysis of raw geological datasets^21^. This model describes the relationships between geological attributes and the cumulative frequency of samples, and is formulated as follows^41–43^.

3 $$\:N\left(\ge\:\rho\:\right)=F{\rho\:}^{-D}$$

where ρ denotes the elemental concentration, N(≥ρ) represents the cumulative number of samples with concentrations greater than or equal to ρ, F is a constant, and D denotes the fractal dimension of the concentration distribution. According to Mandelbrot (1983) and Deng et al. (2010), log–log plots of N(≥ρ) versus ρ typically display straight-line segments with varying slopes (− D), each corresponding to distinct concentration intervals^10,41^ This method is based on the principle that as concentration increases, the number of samples decreases.

#### Number–size model

The number–size (N–S) method, originally proposed by Mandelbrot (1983), is used to describe the distribution of geochemical populations^10^. This model expresses the relationship between the variable of interest and the cumulative number of samples possessing that characteristic^42^. A power-law frequency model for the N–S relationship, based on the frequency distribution of elemental concentrations and the cumulative number of samples with those attributes, is formulated as follows:

4 $$\:N\left(\ge\:\rho\:\right)=K{\rho\:}^{-D}$$

where ρ denotes the elemental concentration, N(≥ρ) represents the cumulative number of samples with concentrations greater than or equal to ρ, K is a constant, and D is the fractal dimension of the concentration distribution. Based on the studies of Deng et al. (2010) and Mandelbrot (1983), log–log plots of N(≥ρ) versus ρ exhibit straight-line segments with different slopes (− D), each corresponding to different concentration intervals^10,41^.

## Results

Using the determined thresholds, the data were divided into separate populations. Numerical validation was then employed to identify the most appropriate clustering approach for this study area. To establish threshold values for class boundaries, log–log plots were generated for each fractal model, where the inflection points indicate thresholds separating different classes. The log–log plots for each fractal model are presented in Fig. 2, and were prepared using QGIS version 3.28.3 (https://qgis.org) based on processed induced polarization chargeability data.

**Fig. 3:**
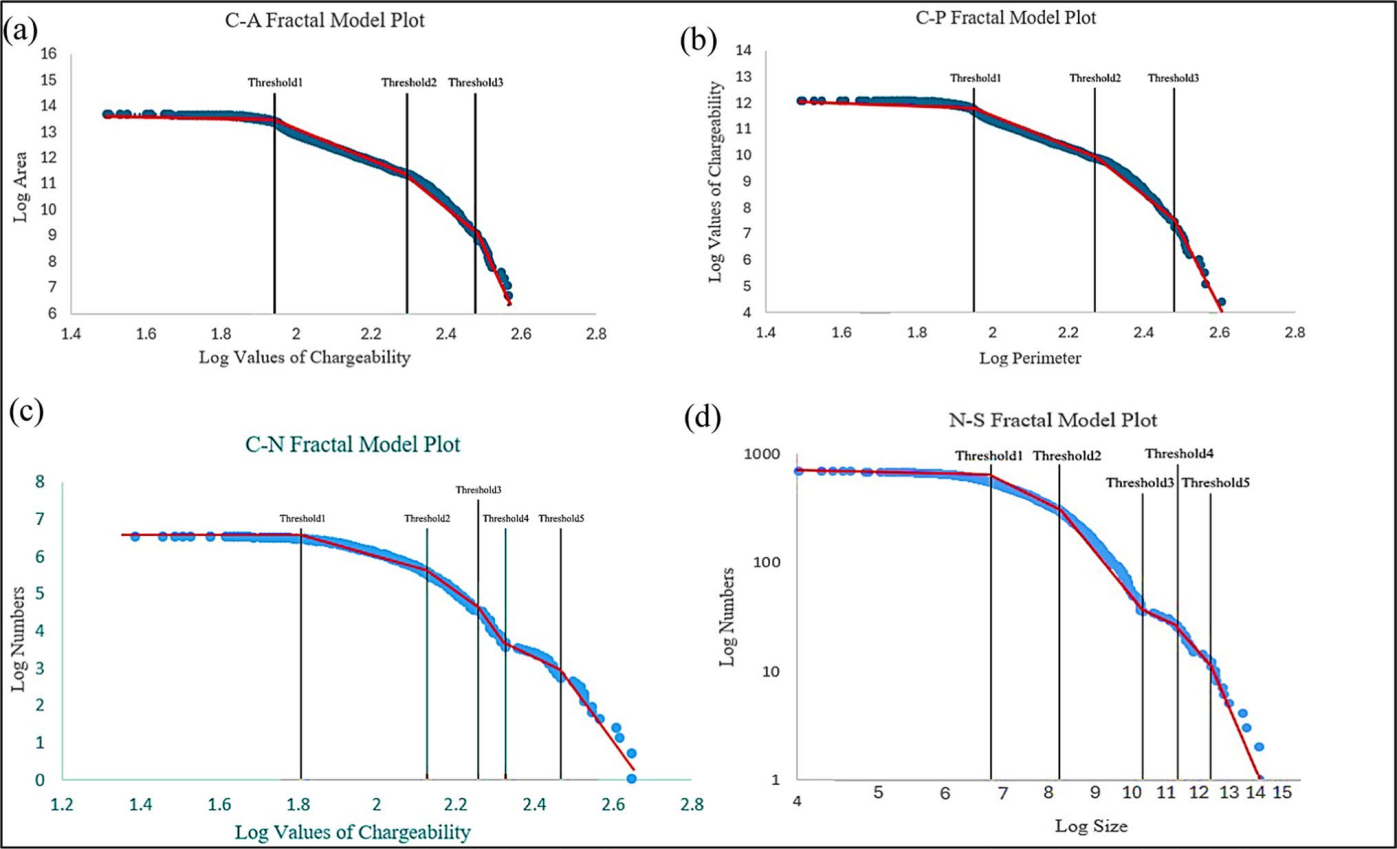
Log–log plots derived from different fractal models for chargeability data: (**a**) Concentration–Area (C–A) model, (**b**) Concentration–Perimeter (C–P) model, (**c**) Concentration–Number (C–N) model, and (**d**) Number–Size (N–S) model. The identified inflection points in each plot correspond to threshold values used for distinguishing anomaly and background populations. Plots were produced using Python version 3.14.0 (https://www.python.org) with Matplotlib and NumPy libraries.

Based on the threshold values determined in each fractal method, the data were classified into four to six distinct populations, and the corresponding chargeability distribution maps were generated for each model. Figure 3 illustrates the spatial distribution of chargeability delineated by the different fractal methods, prepared using Python 3.14.0 (https://www.python.org**)** with Matplotlib and NumPy.

**Fig. 4:**
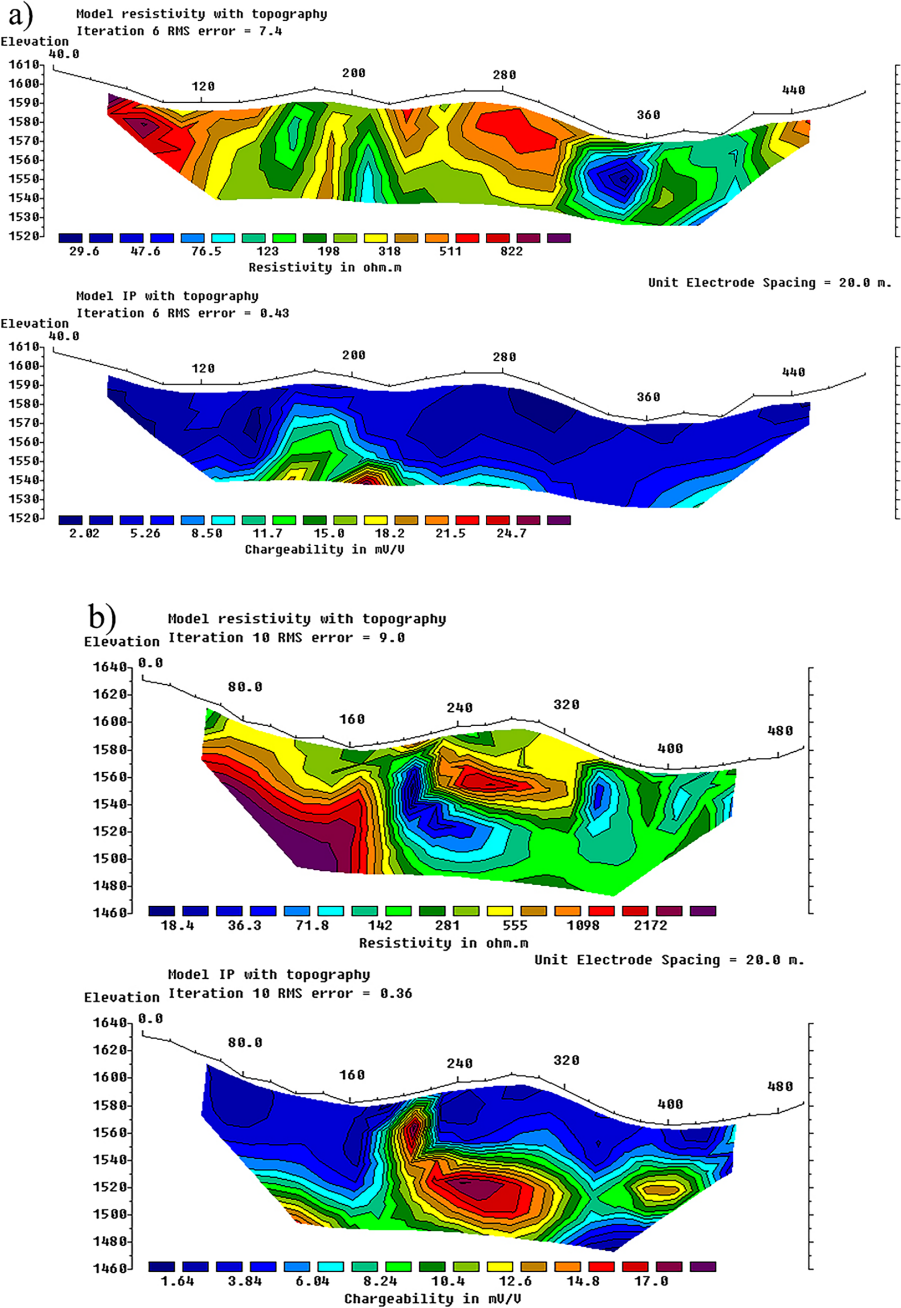

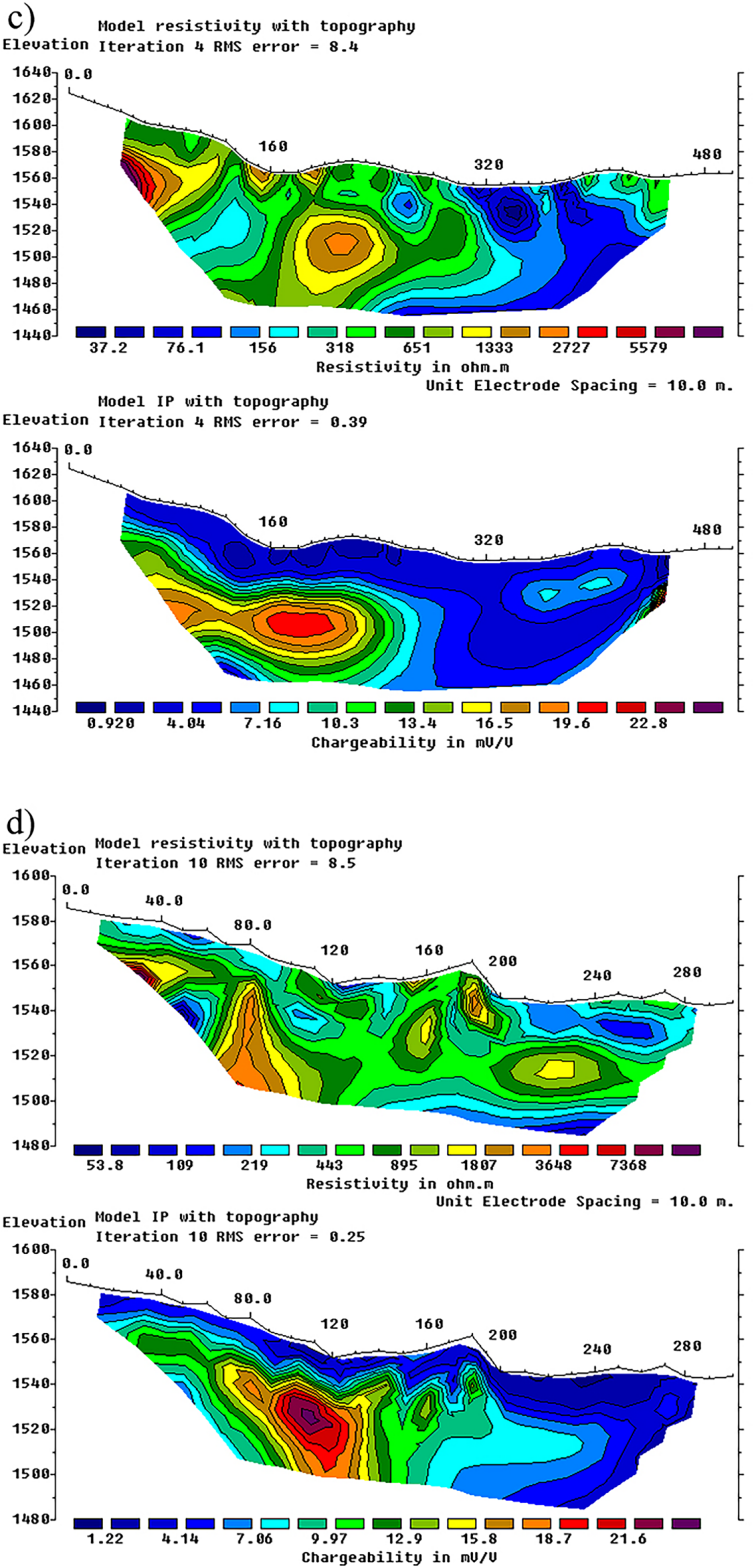
Vertical sections resulting from the inversion of electrical resistivity (top) and induced polarization chargeability (bottom) data. Panels (**a**) to (**d**) correspond to Profiles P1 to P4, respectively, with P1 being the westernmost and P4 the easternmost profiles in the study area (see Fig. 2 for the locations of the profiles). Inversions were performed using RES2DINV version 5.0 (https://www.seequent.com/products-solutions/res2dinv).

The chargeability and resistivity data acquired from the geoelectrical profiles conducted in the study area were inverted following initial processing, and the corresponding vertical sections of resistivity and chargeability were produced for each profile. Figure 4, illustrates vertical sections resulting from the inversion of electrical resistivity (top) and induced polarization chargeability data, carried out using RES2DINV version 5.0(https://www.seequent.com/products-solutions/res2dinv-and-res3dinv/**).**

## Discussion

To compare the fractal analysis methods and evaluate their performance in classifying chargeability data and accurately delineating the geometry and boundaries of anomalies, four validation indices were employed: Silhouette, standard deviation of Silhouette (cluster stability), Davies–Bouldin, and Calinski–Harabasz. The Silhouette index, computed based on Euclidean distance, evaluates how well each data point fits within its assigned cluster compared to other clusters, with values close to 1 reflecting optimal cluster compactness and separation^6^. Among the different fractal clustering models, the C–P method achieved the highest Silhouette index, indicating the best performance. The standard deviation of the Silhouette index, which measures the dispersion or instability in clustering, was also lowest for the C–P model, indicating high clustering stability. The Davies–Bouldin index, which is based on the proximity of cluster members to their centroid and the separation between clusters (lower values are better), again identified the C–P model as optimal. The Calinski–Harabasz index, which considers the similarity of points within clusters and the dispersion between clusters (higher values are better), was also highest for the C–P model^6^ The validation indices for all four methods are summarized in Table 1, with the best values in each metric highlighted in bold. Overall, the validation indices confirm that the Concentration–Perimeter (C–P) fractal model provides the most reliable and robust clustering results for this case study.

**Table 1:** Validation indices for the fractal clustering methods applied to the chargeability dataset. The best values for each metric are highlighted in bold.

Fractal method	Silhouette index	Davies–Bouldin index	Calinski–Harabasz index	Cluster stability (Std of silhouette)
C–A	0.426	0.496	262.582	0.019
C–P	**0.590**	**0.447**	**1279.769**	**0.013**
C–N	0.423	0.511	737.649	0.019
N–S	0.474	0.607	1034.259	0.013

Nevertheless, the interpretation of chargeability anomalies remains influenced by data quality and resolution and subsurface complexity, which should be considered when extending the proposed approach to other exploration settings. Moreover, the relative performance of fractal clustering models may be influenced by geological factors such as lithological heterogeneity, structural complexity, and physical property contrasts associated with mineralization.

Therefore, while the C–P model proved optimal for the Kabudan case study, its performance in other geological settings should be evaluated in relation to local geological and geophysical conditions.

The sections generated from the geoelectrical profile data over the chargeability anomalies confirmed the subsurface continuity of the anomalous zones. In the westernmost profile (P1), the first 140 m correspond to rhyolite and acidic tuff units, which display relatively high electrical resistivity. At distances of 160–200 m and 340–400 m along the profile, two low-resistivity zones were identified, indicative of a conductive environment (Fig. 4a, top). Chargeability data in the deeper section between 160 and 200 m from the profile start reveal a zone of high chargeability, suggesting the presence of a sulfide-bearing mass or vein (Fig. 4a, bottom).

For profile P2, the vertical section shows a distinct lithological boundary at 180 m. Between 180 and 220 m and 340–500 m, a low-resistivity zone with high chargeability is observed at depth (Fig. 4b, bottom). Field observations indicate that the first 140 m of profile P3 are composed of rhyolites with relatively high resistivity, followed by a fault zone between 140 and 200 m, which is also evident in the resistivity section (Fig. 4c, top). Between 140 and 160 m, a surface silicified vein is observed, likely associated with the fault zone; in the resistivity data, a high-resistivity vein beneath this zone likely represents the silicified layer that has been displaced by the fault. High chargeability directly below this zone further supports the potential for mineralization (Fig. 4c, bottom). The 140–200 m interval also coincides with outcropping, silica-rich veins, visible both in the resistivity and chargeability data, confirming the presence of an anomaly in this section.

Borehole data obtained from the main anomaly zone in the western part of the exploration area—whose locations are also shown on the fractal model maps—confirm the reliability of the geophysical models. The specifications of these boreholes are listed in Table 3.

**Table 3:** Specifications of the drilled boreholes in the Kabudan exploration area.

Borehole ID	X (m)	Y (m)	Depth (m)	Dip (°)	Azimuth (°)
BH01	589,177	3,920,891	130	70	172
BH02	589,177	3,920,841	101	70	172
BH03	589,252	3,920,816	109	70	172

**Fig. 5:**
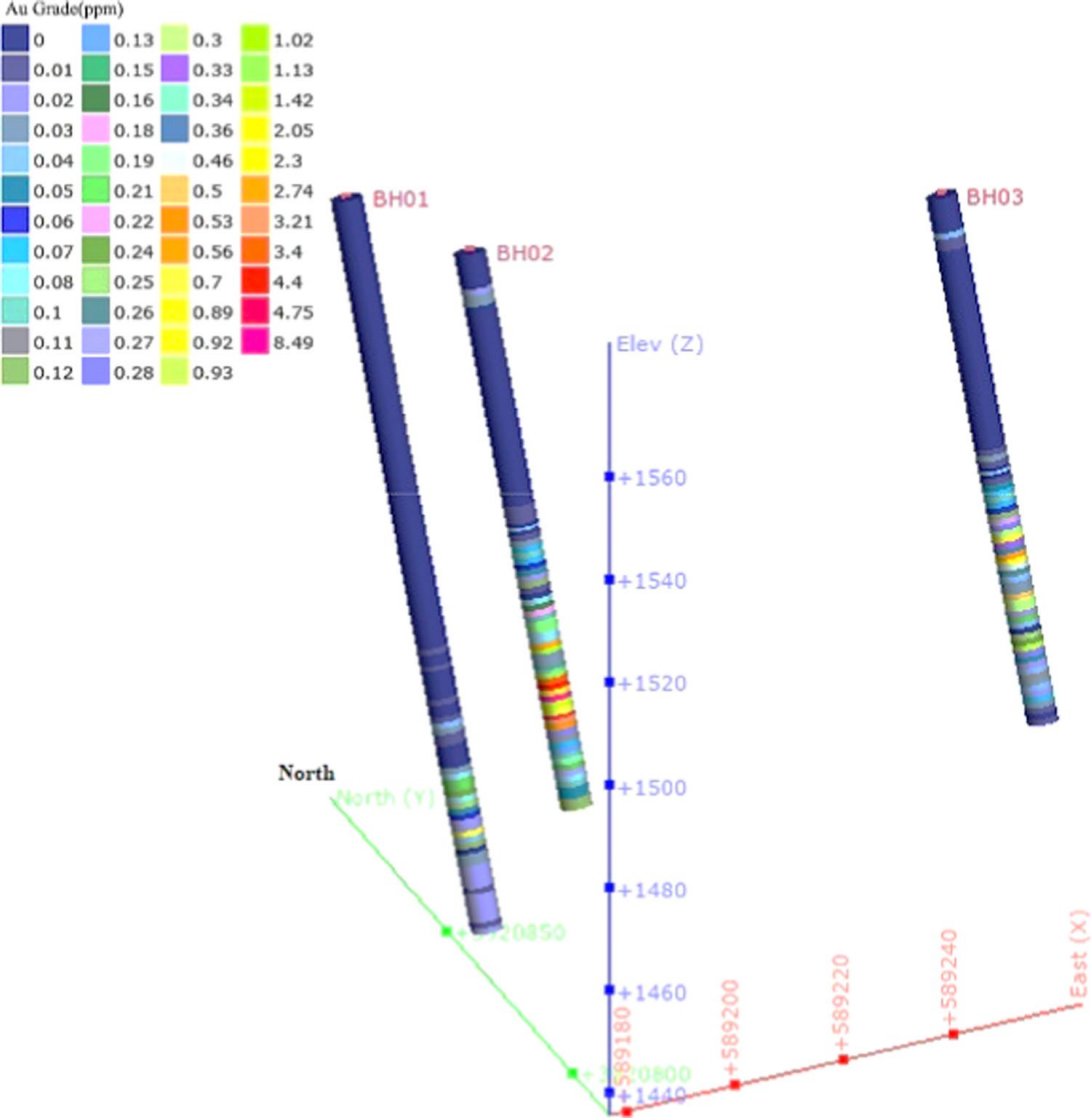
Three-dimensional model of the drilled boreholes with their corresponding assay results, viewed from the southwest. Prepared using Geosoft Oasis Montaj version 8.4 (https://www.seequent.com/products-solutions/oasis-montaj/).

**Fig. 6:**
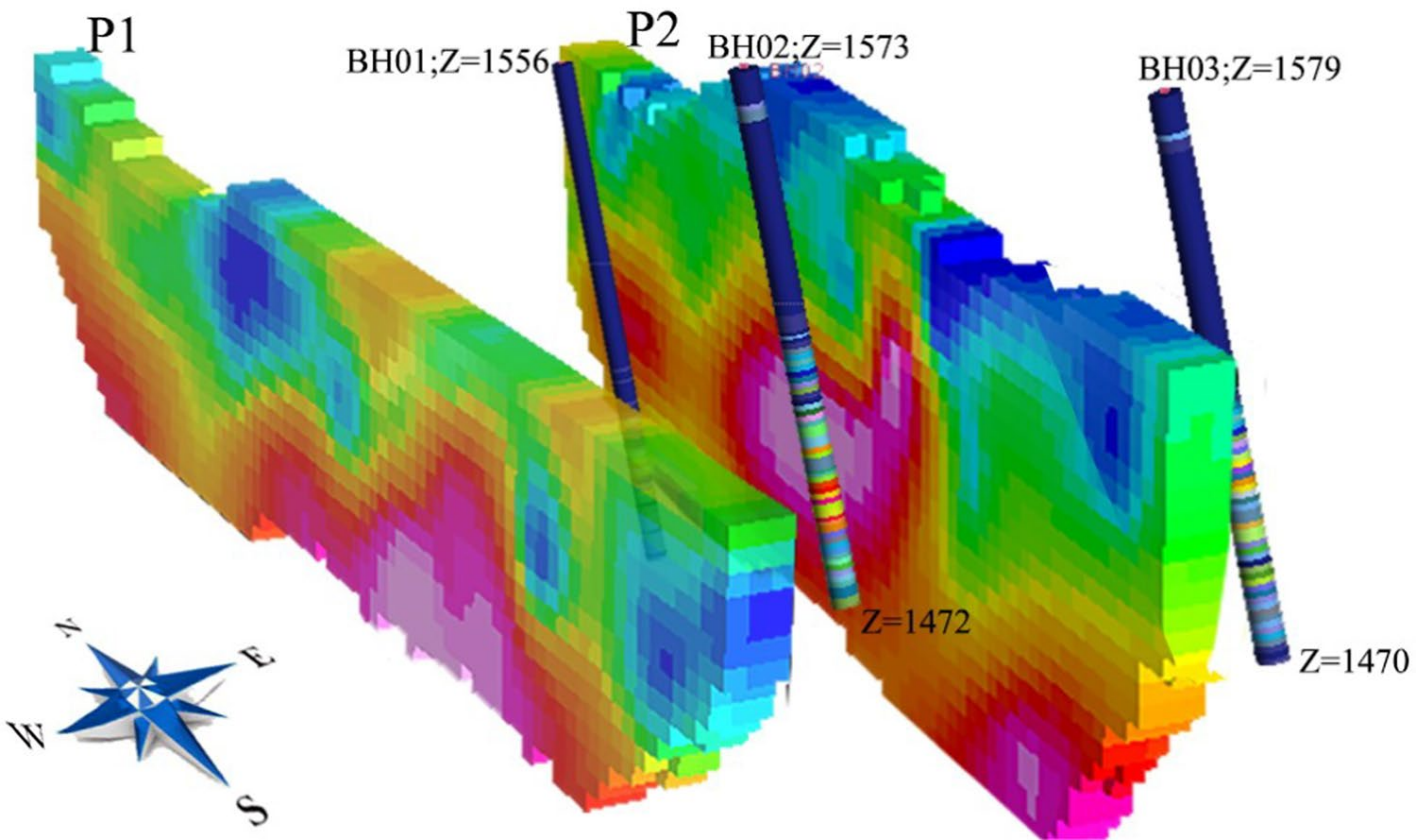
Three-dimensional visualization of borehole locations in relation to the chargeability inversion sections for Profiles P1 and P2. Prepared using Geosoft Oasis Montaj version 8.4 (https://www.seequent.com/products-solutions/oasis-montaj/). Data availability. Data for this study is available from the corresponding author upon reasonable request.

Figure 5 presents a three-dimensional borehole model together with the assay data, while Fig. 6 illustrates the spatial relationship between the boreholes and the main vertical cross-sections (profiles P1 and P2), confirming the consistency between the drilling results and the chargeability inversion data. Both three-dimensional visualizations were prepared using Geosoft Oasis Montaj version 8.4 (https://www.seequent.com/products-solutions/oasis-montaj/).

The lithologies encountered in BH02, which coincides with the chargeability anomaly on profile P2, include pyrite, metamorphosed sandstone, and green schist from 68 to 75 m depth. The highest assay, 8 ppm Au, was recorded at 77–78 m. The high-grade interval (75–83 m) consists of magnetite, pyrite, chalcopyrite, and green schist, showing a strong match between borehole and geophysical anomaly data. The highest gold content observed in BH03, drilled at the eastern end of profile P2, was 2 ppm within sericite–schist and silicified zones, while BH01, located 50 m north of BH02, contained very low gold grades. Thus, combining C–P fractal analysis with IP and inversion results not only enabled the discrimination of surface anomalies, but also demonstrated excellent correspondence with high-grade zones confirmed by drilling.

## Conclusion

This study was conducted to enhance the identification and delineation of mineralized zones in geologically complex settings, through the integrated application of fractal analysis and geophysical inversion in the Kabudan gold exploration area, northeastern Iran.

Initially, induced polarization (IP) data acquired through rectangular array surveys were processed using four well-established fractal clustering models: Concentration–Area (C–A), Concentration–Perimeter (C–P), Concentration–Number (C–N), and Number–Size (N–S). Each model was systematically applied to classify chargeability anomalies and to delineate potential mineralization zones. Subsequently, quantitative validation was performed using four statistical indices—Silhouette, Davies–Bouldin, Calinski–Harabasz, and cluster stability—to evaluate the reliability and consistency of each fractal approach. Among the tested models, the C–P fractal model exhibited the highest clustering quality, reflected by optimal values across all validation metrics.

To confirm the spatial and vertical continuity of the anomalies detected through fractal analysis, two-dimensional inversion of electrical resistivity and IP data was carried out using a robust (L1-norm) algorithm. The inverted sections revealed continuous subsurface anomalies consistent with the clustering results. Furthermore, drill-hole data—showing high-grade sulfide mineralization up to 8 ppm Au in borehole BH02—provided strong geological validation of the interpreted targets.

Collectively, these results demonstrate that integrating fractal clustering with geophysical inversion offers a powerful and quantitative framework for subsurface exploration. The proposed workflow not only enhances anomaly discrimination and target prioritization in structurally and lithologically complex areas but also establishes a transferable and reproducible methodology for other mineral exploration projects. Although the proposed workflow is transferable, its application to other regions should consider variations in geological setting, data quality, and survey design. Future work should extend the fractal clustering framework to three-dimensional geophysical models and integrate machine learning algorithms to refine anomaly detection and classification. Overall, the synergistic combination of fractal analysis, geophysical inversion, and statistical validation provides a robust, cost-effective, and scalable strategy for next-generation mineral exploration. This integrated approach can significantly reduce exploration risk and operational costs, providing mining companies and researchers with a scientifically validated tool for prioritizing drilling targets in challenging geological terrains.

## Data Availability

Data for this study is available from the corresponding author upon reasonable request.
